# Body-size dependent foraging strategies in the Christmas Island flying-fox: implications for seed and pollen dispersal within a threatened island ecosystem

**DOI:** 10.1186/s40462-022-00315-8

**Published:** 2022-04-11

**Authors:** Christopher M. Todd, David A. Westcott, John M. Martin, Karrie Rose, Adam McKeown, Jane Hall, Justin A. Welbergen

**Affiliations:** 1 The Hawkesbury Institute for the Environment, Western Sydney University, Richmond, NSW 2753 Australia; 2 Commonwealth Scientific and Industrial Research Organisation (CSIRO), 47-67 Maunds St, Atherton, QLD 4883 Australia; 3 Atherton, Australia; 4 Taronga Institute of Science and Learning, Taronga Conservation Society Australia, Bradleys Head Rd, Mosman, NSW 2088 Australia; 5 Commonwealth Scientific and Industrial Research Organisation (CSIRO), Waite Rd, Urrbrae, SA 5064 Australia

**Keywords:** Bats, Ecosystem services, Foraging range, Fruit bats, GPS telemetry, Pollination, Pteropodidae, *Pteropus natalis*

## Abstract

**Background:**

Animals are important vectors for the dispersal of a wide variety of plant species, and thus play a key role in maintaining the health and biodiversity of natural ecosystems. On oceanic islands, flying-foxes are often the only seed dispersers or pollinators. However, many flying-fox populations are currently in decline, particularly those of insular species, and this has consequences for the ecological services they provide. Knowledge of the drivers and the scale of flying-fox movements is important in determining the ecological roles that flying-foxes play on islands. This information is also useful for understanding the potential long-term consequences for forest dynamics resulting from population declines or extinction, and so can aid in the development of evidence-based ecological management strategies. To these ends, we examined the foraging movements, floral resource use, and social interactions of the Critically Endangered Christmas Island flying-fox (*Pteropus natalis*).

**Methods:**

Utilization distributions, using movement-based kernel estimates (MBKE) were generated to determine nightly foraging movements of GPS-tracked *P. natalis* (*n* = 24). Generalized linear models (GLMs), linear mixed-effect models (LMMs), and Generalized linear mixed-effects model (GLMMs) were constructed to explain how intrinsic factors (body mass, skeletal size, and sex) affected the extent of foraging movements. In addition, we identified pollen collected from facial and body swabs of *P. natalis* (*n* = 216) to determine foraging resource use. Direct observations (*n* = 272) of foraging *P. natalis* enabled us to assess the various behaviors used to defend foraging resources.

**Results:**

Larger *P. natalis* individuals spent more time foraging and less time traveling between foraging patches, traveled shorter nightly distances, and had smaller overall foraging ranges than smaller conspecifics. Additionally, larger individuals visited a lower diversity of floral resources.

**Conclusions:**

Our findings suggest that smaller *P. natalis* individuals are the primary vectors of long-distance dispersal of pollen and digested seeds in this species, providing a vital mechanism for maintaining the flow of plant genetic diversity across Christmas Island. Overall, our study highlights the need for more holistic research approaches that incorporate population demographics when assessing a species’ ecological services.

**Supplementary Information:**

The online version contains supplementary material available at 10.1186/s40462-022-00315-8.

## Background

Animal-mediated pollination and seed dispersal are fundamental to shaping the ecological and evolutionary processes that affect biodiversity and help maintain ecosystem health. Over ecological timeframes, dispersal methods and patterns influence where individual plants are deposited, their prospects for survival, and ecosystem-wide population dynamics through effects on immigration, emigration and survival [37]. Over evolutionary timeframes, dispersal patterns determine both gene flow between populations and the frequency with which a species experiences new selection regimes that facilitate evolution in novel environments [73]. Among plants that are dispersed by animals, gene-flow through seed dispersal and pollination are affected by various characteristics of a dispersers’ biology such as population density, food availability, and behavior [43, 46]. Despite the recognized importance of the dispersal services provided by animals, the processes that influence seed and pollen dispersal are not fully understood. How an animal’s behavior affects its foraging patterns and movements, and therefore its dispersal of seeds and pollination of flowers, is of particular interest among conservation biologists. This knowledge can be incorporated into models estimating dispersal patterns [88] and assess the importance of the dispersers’ ecological functions. Together this information can be used to predict how anthropogenic disruptions to the dispersal agent may affect the maintenance of diversity and ecosystem health [58].

Foraging movements and associated pollination and seed dispersal services have been shown to vary within species due to a variety of factors [29, 94]. In general, within and between species, larger individuals have a greater capacity to move larger distances than smaller individuals [18, 66, 68]. However, larger individuals also have greater energy demands and a range of behaviors have been documented to secure this energy, including increased foraging area [45, 54]. Social interactions, such as those involved in resource defense can also influence the foraging space of individuals, and so limit or increase the quantity of seed and pollen moved by a given dispersal agent [42]. If an individual is successful at monopolizing a stable resource, the number of other foraging sites it visits is likely to decrease and the quality of its dispersal service is likely to be reduced [42]. Conversely, the foraging interference caused to competitors by a territorial individual may increase the dispersal distances provided by the competitors by forcing them away from the fruiting or flowering tree [44, 46, 53, 71]. In many systems, resource defense is mediated by body size-dependent dominance characteristics [5, 20, 72], with larger more dominant individuals tending to be sedentary while defending the richest resources, and smaller individuals being forced to seek out more marginal habitats [17, 38]. Thus, while larger individuals can have greater capacities to move larger distances, competitive interactions at foraging sites can cause the movements and dispersal services of larger individuals to be more localized than those of smaller individuals.

Flying-foxes of the family Pteropodidae are the world’s largest flying mammals, and provide critical ecological services [4], aided by the taxons’ extreme mobility among foraging and roosting areas [13, 31, 86]. As both pollinators and seed dispersers, flying-foxes are vitally important for the preservation and regeneration of forests, especially on islands that have evolved with depauperate pollinator and seed disperser guilds [25, 30]. Throughout the Pacific, flying-foxes are known to visit at least 92 genera of plants in 50 different families [23, 51]. On some islands, flying-foxes are responsible for 80–100% of the seed rain [25]. However, it has been suggested that the effectiveness of flying-foxes as vectors for plant dispersal is strongly dependent on the their population densities [53], which is of serious conservation concern as many insular flying-fox species are in decline [56, 57].

In flying-foxes, larger, older and more experienced individuals defend territories and displace subordinates from foraging areas [22, 52, 71, 87] using a variety of mechanisms including threatening vocalizations and threatening gestures such as wing spreading or clapping, and chases, fighting and aerial pursuit [14, 60, 71, 81, 89, 90]. When the abundance of flying-foxes declines, resource defense interactions become less common, and so flying-foxes may become functionally extinct, ceasing to be effective dispersal agents long before becoming rare [53]. The reduction and loss of insular flying-fox species exacerbates the vulnerability of island endemic plants that are dependent on a limited suite of pollinators and seed dispersers [24, 25], and so it is important to understand the ecological roles that different-sized individuals have as seed and pollen dispersal agents on islands.

The Christmas Island flying-fox (*Pteropus natalis*) is a medium-sized species of flying-fox limited to Christmas Island in the Indian Ocean, where it is considered to perform critical pollen and seed dispersal services for a broad suite of native plants [39, 80]. It is the only remaining native mammal on Christmas Island [2, 34, 91] following the extinctions during the twentieth century of the Christmas Island rat (*Rattus macleari*), the bulldog rat (*Rattus nativitatis*), the Christmas Island shrew (*Crocidura trichura*), and the extinction of the Christmas Island pipistrelle (*Pipistrellus murrayi*) in 2009 [47]. *Pteropus natalis* has recently undergone a 50 – 75% population decline [64] so that the species is now listed as Critically Endangered under Australian federal legislation [27]. The cause of its decline is not fully understood, which is of serious concern for the conservation of this species and the floral species it services within a small insular ecosystem. *Pteropus natalis* is known to forage on the pollen and fruits of at least 51 species of plants (23 native, 28 introduced) (Additional file 1: Table S1) from 44 genera and 31 families (personal observations, [39, 80]. Several of the endemic plant species are thought to be “chiropterophilous” [39, 80] as they are characterized by large, odorous, nectar rich flowers that open at night [69], further highlighting the important and unique ecological role that *P. natalis* plays as a vector for plant dispersal on Christmas Island.

In this study we examine the foraging ecology of *P. natalis*. Specifically, we document *P. natalis’* foraging movements and diet, and describe their agonistic interactions at foraging sites. Based on our observations and the literature, we predict that larger individuals will i) visit fewer foraging sites, ii) move less frequently between foraging sites, and as a consequence of this, iii) forage on fewer plant species. We consider our findings in light of alternative explanations of how body size may affect foraging movement dynamics, and discuss the implications for the ecological roles that different-sized flying-foxes have as seed and pollen dispersal agents on islands.

## Methods

### Study location

Christmas Island is a small, 135 km^2^, geographically isolated island in the Indian Ocean, located approximately 380 km south of Java, Indonesia, located at 10° 25′ S and 105° 40′ E. It is monsoonal with distinct wet (November–June) and dry (July–October) seasons. The vegetation on Christmas Island has been categorized into seven main types: evergreen forest, semi-deciduous forest, deciduous scrub, perennial wetland forest, coastal fringe, rehabilitation, regrowth and weed dominated, and pioneer growth (see Additional file 1: Table S2 for further classification and habitat description).

### Data collection

#### Capture and body measurements

**Fig. 1:**
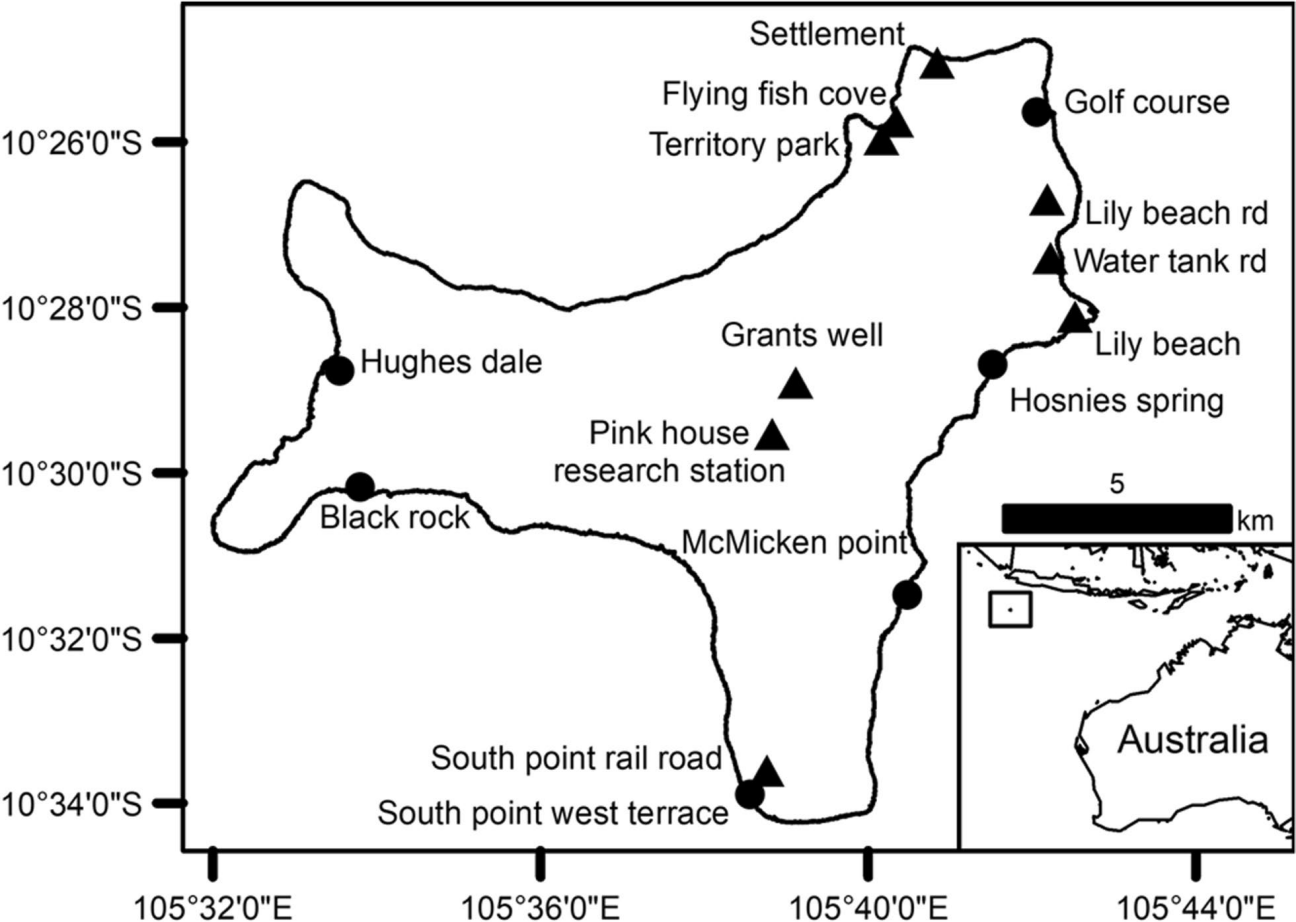
The locations of nine foraging (▲) and six roost (●) sites on Christmas Island where *Pteropus natalis* were captured during this study between August 2015 and November 2017

*Pteropus natalis* were caught at foraging and roost sites across Christmas Island (Fig. 1) between August 2015 and November 2017 using 3.2 × 12 m nylon mist nets with a 45 × 45 mm mesh size (Ecotone; Gdynia, Poland) or an aluminum anglers’ landing net with a 64 × 56 cm hoop dimension mounted on an 8 m pole. Flying-foxes were weighed to the nearest 1 g using a 5-kg digital scale (Breville, Sydney, New South Wales, Australia). Forearm length, thumb length, thumb claw length, and tibia length were measured to the nearest 0.1 mm with calipers (Just Tools, Melbourne, Victoria, Australia).

#### Pollen collection and identification

Pollen was collected from captured *P. natalis* by swabbing a cotton ball once across the face, body, and both sides of the wings. Pollen samples were then stored in 90% ethanol. Pollen samples (*n* = 216) were collected from individuals captured between 1400 and 0730 h. The greatest proportion (50%) of pollen samples was collected from individuals captured between 1700 and 2100 h (see Additional file 1: Figure S1). We removed samples (*n* = 46) collected between the months January to June (a period when flowering is at its lowest) because they did not contain any pollen. We also removed samples (*n* = 55) containing pollen loads < 20 total pollen grains, that were substantially lower the mean pollen load ($\overline{x }$ = 150.89). Neither the time of capture nor nightly rainfall was a significant factor in the presence or absence of pollen among the collected samples (GLMM; time of capture: *Z*_4,216_ = 0.32, *P* = 0.751; rainfall: *Z*_4,216_ = − 1.62, *P* = 0.105).

To better understand the foraging resources used by *P. natalis* we identified the pollen collected from facial and body swabs among the broader population (*n* = 115). At the time of capture, a cotton ball wiped over the face, wings and fur of an individual animal was saturated in ethanol until processing. To extract the pollen samples, the cotton ball was placed into a 300 ml glass beaker with 40 ml of deionized water and then placed into an ultrasonic bath and agitated for several minutes to remove the pollen from the cotton. The cotton was removed and 5–6 drops of 1% Safranin stain solution (Thermo Fisher Scientific Pty Ltd, Waltham, Massachusetts, United States) was placed into the pollen suspension. A portion of the pollen suspension was then placed in a 10 mL conical centrifuge tube and centrifuged at 3000 RPM for 3 min. The supernatant was decanted without disturbing the pollen collection at the bottom of the tube. The pollen pellet was then resuspended using a vortex mixer and the centrifuge tube was refilled with remaining pollen suspension and placed back in the centrifuge. This process was repeated until all of the pollen suspension had been centrifuged and poured off leaving a single pollen pellet in the conical tube. A glass Pasteur pipette was used to extract the pollen pellet and place it into a glass vial with glycerol for storage. A 20 μl aliquot of the pollen/glycerol mixture was placed onto a glass slide, covered with a 22 × 40 mm coverslip, and sealed with lacquer. Pollen was viewed at 400 × magnification using a Zeiss Axio Imager 1 microscope (Carl Zeiss Microscopy Pty Ltd, Jena, Germany) and identified to genus level, and species level where possible, using The Australia National University Australasian Pollen and Spore Atlas (http://apsa.anu.edu.au) and reference samples collected on Christmas Island. While the different pollen types all represent distinct species, we use the term “pollen type” to account for the fact that that a small proportion of pollen types could not be formally classified to species level. Three transects were made across the length of each slide, counting the numbers of each pollen type identified.

#### GPS telemetry

We deployed solar-powered Camazotz GPS telemetry nodes [41], hereafter "GPS nodes", on 24 of the caught flying-foxes (16 males, 8 females; Additional file 1: Table S3). Each GPS node was 5 × 3 × 1.5 cm, plus a 6 cm antenna. GPS nodes were fixed with metal rivets to a collar that consisted of a 117 × 7.5 × 1 mm piece of leather sewn to a 110 × 15 × 2 mm piece of neoprene. A 3.75 mm neoprene overlap was allowed on the linear sides of the leather to avoid it contacting the skin and causing discomfort for the flying-fox. The collar, with the attached GPS node, was trimmed to fit individual flying-foxes and secured using monofilament polyglyconate absorbable sutures (Covidien, Dublin, Ireland). Sutures were placed at the top and bottom of the overlapping piece of leather. To aid in the relocation of the tagged flying-fox we attached a 1 g VHF transmitter (ATS Telemetry, Isanti, Minnesota, United States) to the leather of the collar next to the GPS node. The combined weight of the GPS node, VHF transmitter, and collar was 19 g; less than 5% of each animal’s body mass.

We tracked the 24 individuals (16 male and 8 female) for 3–28 consecutive nights (1600–0600 h), recording a total of 5992 positions over 253 bat nights (Additional file 1: Table S3). GPS nodes were deployed at nine locations and in eight separate months over the course of the study (Additional file 1: Table S3). GPS nodes were scheduled to record positions every 20 min when battery voltage was between 4 and 3.8 V, every 1 h when voltage was between 3.8 and 3.4 V, and every 12 h when voltage had fallen to between 3.4 and 3.0 V. The daily solar charge was not strong enough to fully re-charge the GPS battery, and once the battery voltage dropped to a lower recording schedule it did not return to the previous, more frequent, GPS fix recording rate. Therefore, data acquisition rates were chosen to optimize the frequency and accuracy (~ 5 m) of recorded positions. Recorded positions were downloaded from the GPS nodes via a remote base station [41] within a range of < 200 m. (These data are available in movebank.org; study name: “Movements of the Christmas Island flying fox, Australia”.).

To minimize stress while fitting the GPS nodes all flying-foxes were anesthetized with vaporized isoflurane (Ohmeda Tec 4, Avon, Ohio, United States; [40] at an induction rate of 5% and maintenance rate of 1.5%, delivered in 1 L/min oxygen. Our research protocols followed the American Society of Mammalogists guidelines for research on live mammals [75], and were approved by the Animal Care and Ethics Committee of Western Sydney University (Project Protocol No. A11140). Permits to capture and process *P. natalis* were issued by the Christmas Island National Park (Permit No. CINP-2015-6-1).

### Foraging movement and habitat use modeling

Movement-based kernel estimates (MBKE; [10] were created based on the GPS node data using the AdehabitatHR package (version 0.4.15; [19] in program R version 4.0.5 [70]. To identify areas with varying degrees of use, we generated utilization distributions (UD) using the biased random bridge function [10] in the AdehabitatHR package. We generated 95% UDs to determine an individual’s overall foraging range (FR). We use the term ‘foraging range’ that is defined by Bonaccorso et al. [12] as “the total area traversed by an individual as it searches for food and feeds, as well as movements between day roosts and foraging sites”. To identify and quantify the number of foraging areas (FA) that were intensively and/or repeatedly used within the FR we generated 30% intensity distributions (ID, [11]. The minimum distance between foraging areas was 15 m. We then counted the number of unique FAs visited within a night and across all nights. To further understand the potential dispersal distances provided by *P. natalis,* we calculated the distance from the roost site to all FAs visited within a night, the distance traveled between nightly used FAs, total nightly foraging dispersal distances (i.e., the distance between the furthest two GPS fixes within a night) and the long axis across the entire FR (LAX; i.e., the distance between the furthest two GPS fixes across all nights) (for a complete list of spatial measurements see Table 1). For calculations of FR, and FA we only used GPS fixes captured within the 20 min sampling interval. GPS fixes captured within the 20 and 60 min sampling intervals were used to calculate nightly dispersal distances and the number of nightly FAs visited. GPS fixes captured within the 60 min sampling interval were only used in determining the number of FAs visited when they visited a previously identified FA calculated using the 20 min sampling data. Nights with < 14 GPS fixes were excluded from calculations of nightly dispersal distance and the number of nightly FAs visited. All GPS fixes across all tracking nights were used to calculate LAX (see Additional file 1: Table S3). MBKE polygon data and foraging locations from GPS nodes were imported into ArcGIS v10.3.1 and overlaid onto a Christmas Island Vegetation and Clearing Map [33] at a 2 m resolution. Land-cover categories were assessed for the FR and FA of each tracked individual, identifying the number of unique vegetation habitats within each FR and FA (Additional file 1: Table S2). Over the course of this study, we were successful in tracking multiple individuals with overlapping FAs during three separate periods (Additional file 1: Table S4). For each group of simultaneously tracked individuals we calculated the area among overlapping FAs using the ‘intersect’ tool in ArcGIS (v10.3.1), and provide the percentage of overlap for each overlapping dyad. We also measured the distance between foraging locations for GPS fixes recorded with same timestamp when one or more of the simultaneously tracked individuals occupied the overlapping area of the two FAs.

**Table 1:** Abbreviation, method of calculation, and definition or purpose for interpretation for the spatial measurements collected from 24 Christmas Island flying-foxes (*Pteropus natalis*) fitted and tracked with GPS telemetry nodes between August 2015 and November 2017

Measurement	Abbreviation	Calculation	Definition or purpose
Foraging range	FR	95% utilization distributions (UD)	The total area traversed by an individual as it searches for food and feeds, as well as movements between day roosts and foraging sites
Foraging area	FA	30% intensity distributions (ID)	Identify and quantify the number of intensively and/or repeatedly used foraging areas within the foraging range
Long axis across the foraging range	LAX	Distance between the furthest two GPS fixes across all nights	The scale of movements during the entire tracking period
Total nightly foraging dispersal distance	Nightly dispersal	Distance between the furthest two GPS fixes within a night	The scale of movements during a single night
Distance from roost to foraging areas	Roost to FA	Distance between the roost site and each nightly used foraging area	The scale of movements individuals travel from the roost site in search of food
Distance between foraging areas	Between FA	Distance between each nightly used foraging area	The scale of movements individuals travels between foraging areas in search of food

#### Assessing foraging resource defense

An assessment of agonistic interactions between *P. natalis* individuals at foraging sites was conducted for the purpose of establishing that *P. natalis* engages in foraging resource defense. In all but one observation (see Additional file 2) the body mass and morphological measurements of all individuals observed was unknown. Therefore, we were unable to test for the effects of body size on resource defense. Interactions of foraging *P. natalis* were observed during one to three-hour long surveys (*n* = 272), totaling 306 observation hours. Behaviors exhibited in the defense of foraging resources comprised: (1) ‘scent marking’, whereby an individual rubs its scapular glands along the petioles of leaves or branches; (2) ‘threat display’, whereby an individual directs loud vocalizations, wing spreading, rapid swinging of the thumb claws, at another individual; (3) ‘chase’, whereby an individual aggressively advances towards another individual in a threatening manor; and (4) ‘escalated fights’, whereby an individual uses its wings, claws, and teeth in direct physical contact with another individual (for further details refer to [49].

The size of an individual’s defended patch was estimated by measuring the canopy of known foraging locations using aerial imagery in Google Earth Pro v7.3.3.7786 (Google, Mountain View, California, United States).

### Statistical analysis

All statistical tests were two-tailed, employed an α value of 0.05, and were conducted in R version 4.05 [70]. Continuous variables are characterized by median and interquartile range (IQR). Generalized linear models (GLMs) were constructed with the ‘stats’ package to assess how intrinsic (body mass, skeletal size, and sex) and extrinsic (rainfall, time of year, and the number of GPS fixes) variables affected differences in the size of FR, FA, LAX and the number of vegetation habitats within the FR and FA. Linear mixed-effect models (LMMs) were constructed with the ‘lme4’ package [9] to assess the distance traveled between roost sites and nightly FAs, the distance traveled between nightly used FAs, and total nightly foraging dispersal distances. A Poisson generalized mixed-effect model (GLMM) with a log-link function was constructed with the ‘lme4’ package to assess the number of nightly foraging sites visited, and a Poisson GLM with a log-link function was constructed to assess the diversity of floral resource visited. A single metric for skeletal size was estimated using the score values from a principal component analysis (PCA) on the morphological measurements of forearm length, thumb length, claw length and tibia length (see also; [85]). For all LMM and GLMM models the identity of the individual was included as a random effect to account for repeated measures. The response variables in each of the constructed GLM and LMM models sets were not normally distributed. To normalize the response variable in each of the constructed model sets, excluding the Poisson GLM, an Ordered Quantile (ORQ) normalization transformation was applied to the response variables FR, FA, LAX, distance from roost to FAs, distance between FAs, nightly dispersal distances, and foraging distance between simultaneous tracked individuals. All response variables were transformed using the ‘bestNormalize’ package [67]. The explanatory variables body mass and rainfall were rescaled using the default scale function in R. Pearson’s correlations were used to check for collinearity between explanatory variables. We chose to remove ‘the number of nights tracked’ from all models as it was highly correlated to ‘the number of positions recorded’ (*r* = 0.94, *P* < 0.001). For models predicting the number of pollen types found on *P. natalis*, which represents the foraging behaviors of the broader population (i.e., juveniles, sub-adults and adults of both sexes), ‘skeletal size’ was removed as it was highly correlated to ‘body mass’ (*r* = 0.70, *P* < 0.001). For all other models involving GPS tracked individuals there was no correlation between body mass and skeletal size (*r* = 0.25, *P* > 0.232) or time of year and rainfall (*r* = 0.06, *P* > 0.795). The full models predicting FR, FA, LAX, distance from roost to FAs, distance between FAs, nightly dispersal distances, and the number of pollen types included interactions for body mass and sex, body mass and rainfall, skeletal size and sex, and body mass and time of year (Additional file 1: Table S5). The full model for the number of pollen types included the additional variables time of capture, age class, and interactions for body mass, age class, and sex. Candidate models were compared using Akaike Information Criterion for small sample sizes (AIC_c_). We considered all models within ΔAIC_c_ ≤ 2 (Additional file 1: Table S5) to be competitive and were then averaged [3] using the ‘MuMin’ package [8]. The proportion of variance explained by the fixed effects retained in each of the models was considered by computing the R^2^ values using the ‘rsq’ package for each GLM [93] and the ‘partR2’ package for each LMM and GLMM [77].

## Results

### Foraging ranges

The median foraging range (FR) for all 24 tracked *P. natalis* was 36.27 ha (IQR 14–329 ha) (Additional file 1: Table S6). This included an adult male with a body mass of 374 g, that was active over a much larger area (1936 ha) than any other individual. The smallest FR (4.13 ha) belonged to an adult male with a body mass of 515 g (Additional file 1: Table S6).

The median long axis across the foraging range (LAX) was 2.83 km (IQR 0.70–5.10 km) (Additional file 1: Table S6). The median nightly distance traveled from roost site to foraging area across all individuals was 216.01 m (IQR 62.73–673.18 m). The median nightly distance traveled between FAs across all individuals was 245.99 m (IQR 107.20–627.97 m). The median nightly foraging distance across all individuals and all nights was 0.54 km (IQR 0.23–0.91 km). The longest nightly foraging distance among all individuals and nights was 8.89 km by an adult male weighing 374 g, and the shortest was 3.1 m by an adult female with a body mass of 459 g.

The median foraging area (FA) was 1.07 ha (IQR 0.60–8.13 ha; Additional file 1: Table S6), which represented 3% of the median FR. Over multiple foraging nights, 54% (*n* = 13) of tracked individuals foraged at 5 – 10 FAs, while 21% (*n* = 5) foraged at < 5 FAs, and 25% (*n* = 6) foraged at > 10 FAs (max = 17, Additional file 1: Table S6), and this was not influenced by the number of GPS fixes (LMM; R^2^ = 28.15%, *Z* = 1.43, *P* = 0.153; supplemental material 1: Table S7). Within a single foraging night, 50% (*n* = 12) of *P. natalis* foraged at multiple FAs each night and 46% (*n* = 11) foraged at multiple FAs on 70–92% of all foraging nights. Only one individual foraged at a single FA on 73% of all foraging nights (Additional file 1: Table S6).

**Fig. 2:**
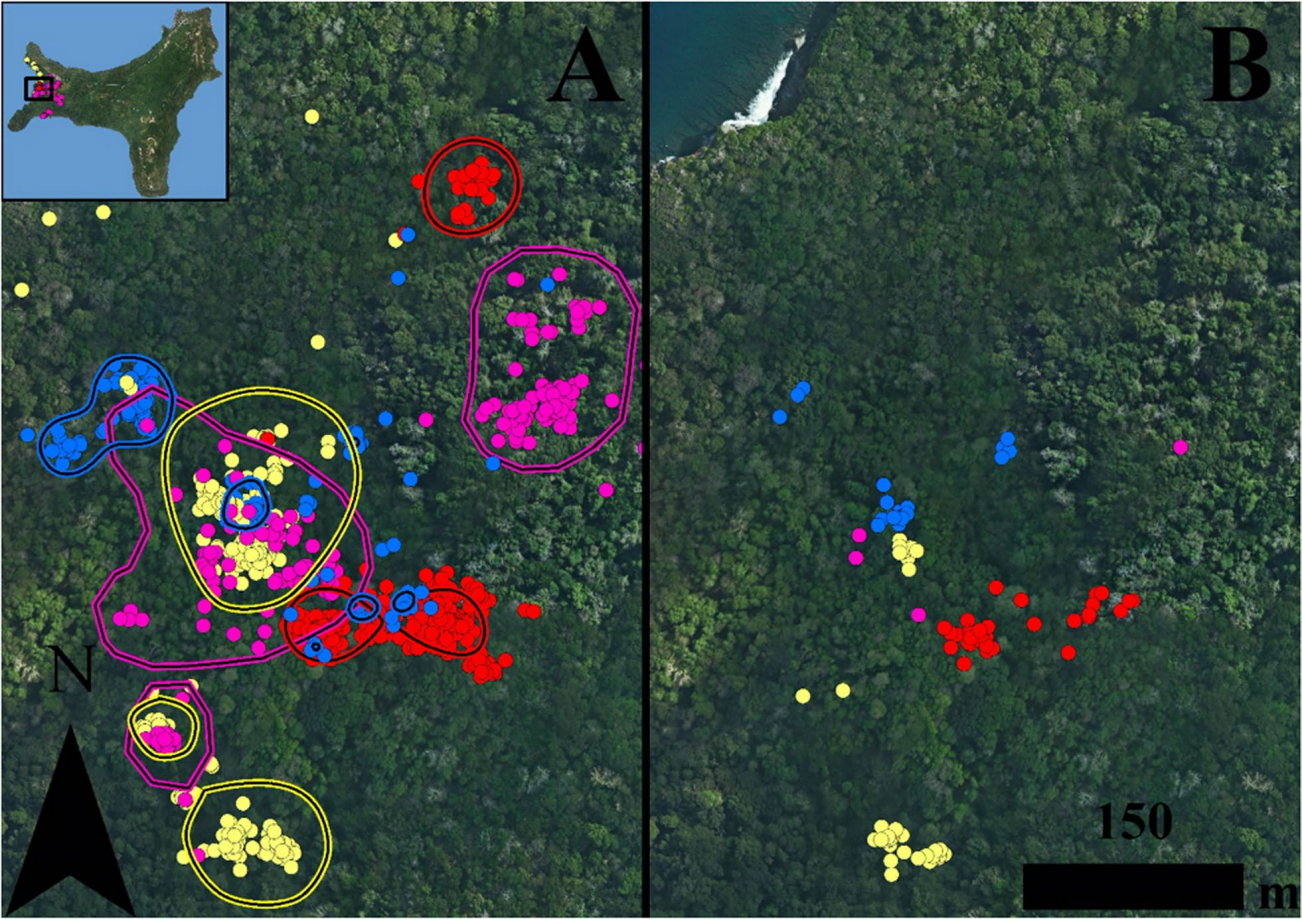
Nightly foraging locations and foraging areas (FAs) of adult female 486 (  ), adult female 840 (  ), adult female 777 (  ) and adult male 806 (  ) *Pteropus natalis* simultaneously tracked with GPS telemetry nodes at Hughs Dales. These data show (A) the foraging locations and foraging areas (FAs) over the course of four nights (26 – 29 October 2017) demonstrating the overlap in foraging areas, and (B) spatially disjunct foraging locations over the course of a single night (29 October 2017)

Foraging areas (FA) of individuals that were tracked simultaneously over the course of each tracking period overlapped by between 0.96 and 58.33% across all tracking nights (Additional file 1: Table S4), suggesting a shared use of foraging resources (Fig. 2a). Only two adult females tracked at Hughes Dale in October of 2017 showed no temporal or spatial overlap among their FAs (Fig. 2a; Additional file 1: Table S4). However, within a single foraging night, FAs were, for the most part, spatially disjunct (Fig. 2b). During periods in which one of the simultaneously tracked individuals occupied the overlapping space of another individuals’ FA, the median distance between foraging locations was 81.37 m (IQR: 44.75 – 262.01 m). There were two adult females (body mass = 432 g and 466 g) who foraged within 5 m from each other in a large patch of bananas over a period of three nights (Additional file 1: Table S4); however, foraging at this area was dominated by the heavier female.

### Effects of body mass on patterns of movements

**Fig. 3:**
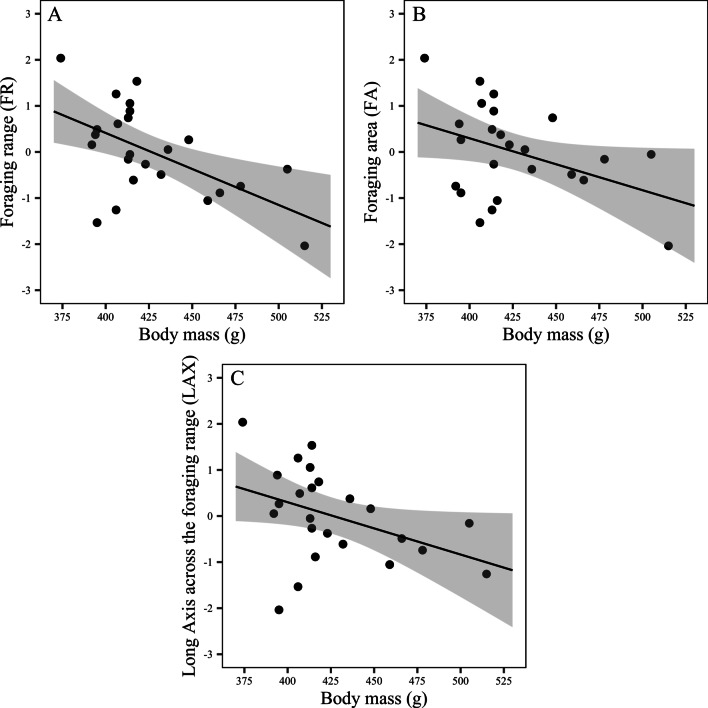
Linear regressions with 95% confidence intervals showing the relationships between body mass and **a** foraging range (FR), **b** foraging area (FA), and **c** long axis across the foraging range (LAX) for 24 Christmas Island flying-foxes (*Pteropus natalis*) tracked with GPS telemetry nodes between August 2015 and November 2017. An Ordered Quantile (ORQ) normalization transformation was applied to the response variables FR, FA, and LAX

Across all individuals, body mass was negatively correlated to the size of their FR (GLM; R^2^ = 46.19%, *Z* = 3.43, *P* < 0.001: Fig. 3a), FA (GLM; R^2^ = 33.31%, *Z* = 2.45, *P* < 0.014: Fig. 3b), and LAX (GLM*;* R^2^ = 33.15%, Z = 2.59, *P* < 0.009: Fig. 3c; Additional file 1: Table S7). Rainfall was also negatively correlated with LAX (GLM; R^2^ = 15.49%, *Z* = 2.01, *P* < 0.044; Additional file 1: Table S7).

**Fig. 4:**
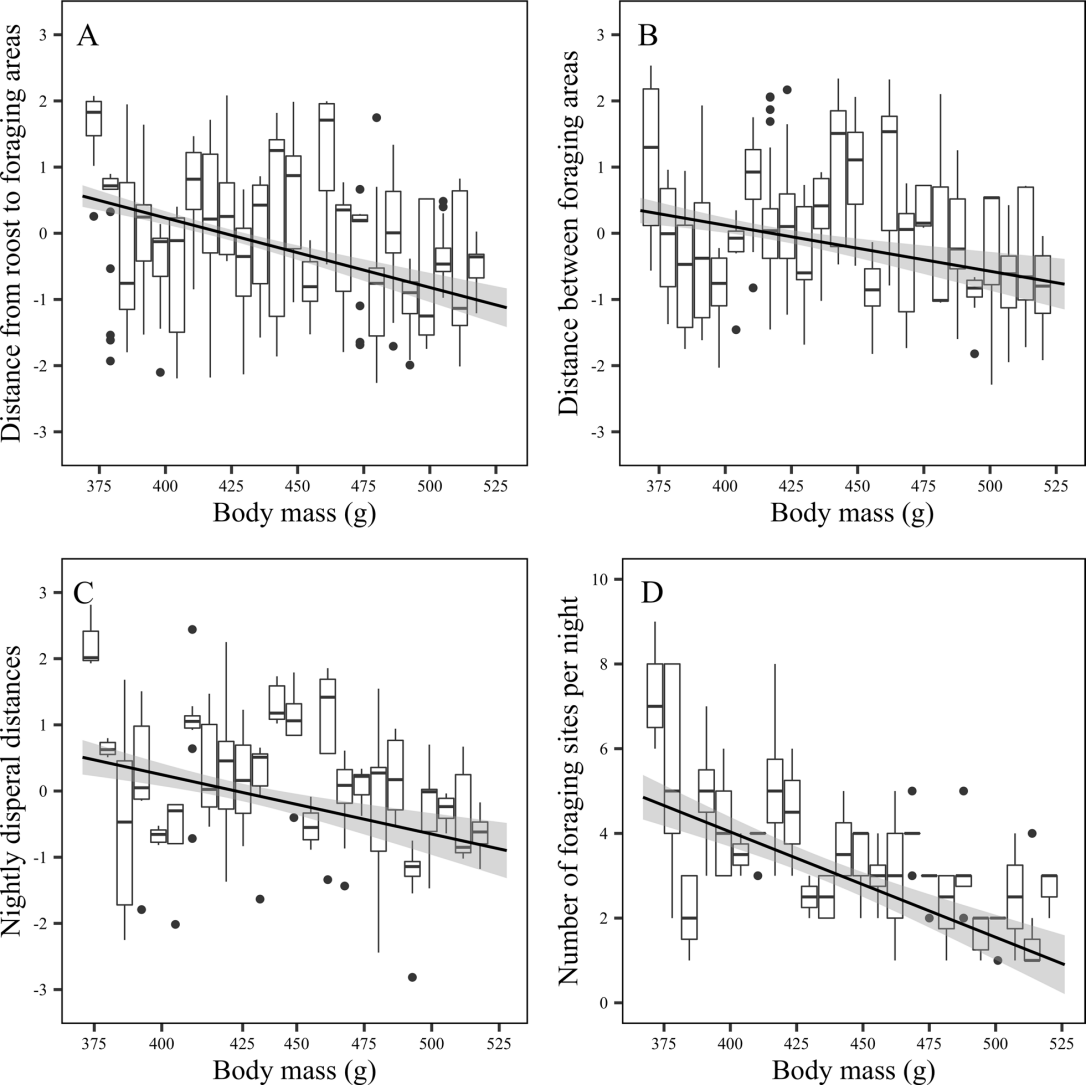
Box-plots identifying the median, lower (Q1) and upper (Q3) quartiles, whiskers (IQR*1.5), and outliers fitted with a linear regression and 95% confidence interval for: **a** distance traveled from roost site to each nightly used foraging areas (FAs) with an Ordered Quantile (ORQ) normalization transformation versus body mass, **b** distance traveled between nightly used foraging areas (FAs) with an (ORQ) transformation versus body mass, **c** total nightly dispersal distances with an (ORQ) transformation versus body mass, and **d** the number of foraging sites visited per night versus body mass for 24 Christmas Island flying-foxes (*Pteropus natalis*) fitted and tracked with GPS telemetry nodes between August 2015 and November 2017

Similar to LAX, the nightly distance traveled between roost site and FAs (Fig. 4a), the distance traveled between nightly used FAs (Fig. 4b), and the nightly foraging distances (Fig. 4c), decreased significantly with increased body mass (LMM; roost to FA: R^2^ = 33.40%, *Z* = 2.95, *P* < 0.007; between FA: R^2^ = 33.99%, *Z* = 3.11, *P* < 0.005; nightly dispersal: R^2^ = 43.44%, *Z* = 2.57, *P* < 0.010; Additional file 1: Table S7). Additionally, the number of FAs visited by *P. natalis* over the course of a single night (Fig. 4D) was negatively correlated with body mass (GLMM; R^2^ = 26.93%*, Z* = 4.97, *P* < 0.001; Additional file 1: Table S7).

The number of vegetation habitats within FRs and FAs significantly increased with the size of an individuals’ FR and FA (Pearson’s correlation; FR: *r* = 0.98, *n* = 24, *P* < 0.001; FA: *r* = 0.77, *n* = 24, *P* < 0.001) and decreased significantly with body mass for FR (GLM; R^2^ = 45.79%, *Z* = 3.29, *P* < 0.002) and FA (GLM; R^2^ = 14.68%, *Z* = 2.47, *P* < 0.014; Additional file 1: Table S7).

Sex was only retained (ΔAIC_c_ ≤ 2) in models predicting the number of pollen types (Additional file 1: Table S5) but was not found to be significant (GLMM: R^2^ = 2.19%, *Z* = 1.69, *P* > 0.090) after model averaging (Additional file 1: Table S7). Sex was not retained (ΔAIC_c_ ≤ 2) in any of the models constructed to explain patterns of foraging movements (Additional file 1: Table S5).

### Floral diversity

**Fig. 5:**
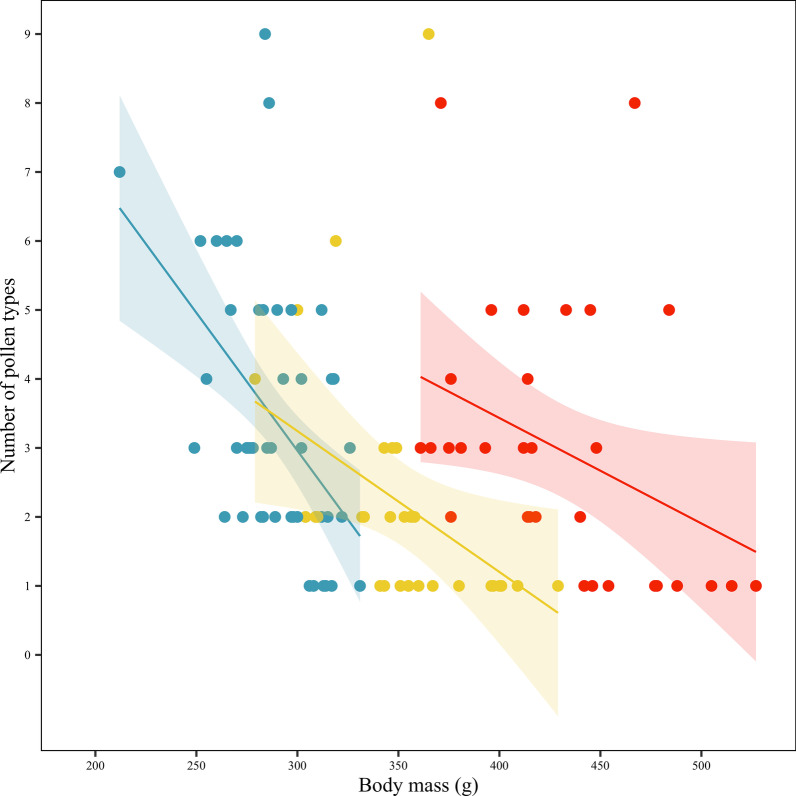
Linear regression with 95% confidence intervals showing the relationship between body mass and the number of pollen types for juvenile (blue, *n* = 53), sub-adult (yellow, *n* = 31) and adult (red, *n* = 31) Christmas Island flying-foxes (*Pteropus natalis*) captured between August 2015 and November 2017

The examination of pollen among all captured *P. natalis* (*n* = 115) revealed that individuals had, on average, pollen from 2.94 (SD = 1.92) plant species, with some individuals having pollen from as many as nine plant species. Within each of the age classes, on average, juveniles had pollen from 3.38 (SD = 1.87) plant species, sub-adults had pollen from 2.97 (SD = 1.94) plant species, and adults had pollen from 2.16 (SD = 1.77). The number of pollen types was negatively correlated with body mass in each of the juvenile, sub-adult, and adult age classes (GLM: R^2^ = 26.52%, *Z* = 3.671, *P* < 0.001; Fig. 5, Additional file 1: Table S7). The number of pollen types was also negatively correlated to body mass in the subset of individuals (*n* = 17) that were tracked and from which pollen counts were available (GLM: R^2^ = 46.03%, *Z* = − 2.41, *P* < 0.017).

### Foraging defense

Over the course of 272 surveys of flying-foxes at foraging sites, we observed 105 instances of foraging resource defense between *P. natalis* individuals. The most frequent defensive behavior observed was vocalization, followed by wing spreading, fighting and chasing. We also observed one instance of scent marking branches by a male *P. natalis* on *C. papaya* (see Additional file 2 for a detailed description of the foraging resource defense behaviors observed). Among smaller trees (e.g., *C. papaya* and *Musa* spp.), *P. natalis* for the most part defended the entire tree, although it was not unusual to see a second individual on the outskirts of the trees’ branches when the canopy volume was > 3 m^3^. Among larger foraging trees (e.g., *Magnifera* spp. and *Ficus* spp.) multiple individuals could be seen, each defending a patch, approximately 3 m^3^, with multiple juveniles on the periphery of the tree.

## Discussion

In this study we found that morphological characteristics, primarily body mass, were linked to *P. natalis*’ foraging movements. Overall, smaller individuals traveled longer distances each night, visited more foraging sites each night, and had larger foraging ranges overall, than larger conspecifics. Smaller individuals also had pollen from a higher diversity of plant taxa on their fur. Combined, these observations suggest that, individually, smaller *P. natalis* provide longer distance pollen dispersal services to a broader range of plant species on Christmas Island than do their larger counterparts. Behavioral observations further indicated that *P. natalis* engages in foraging resource defense, and given that in territorial species, larger individuals have a clear advantage in obtaining and defending territories [76], this provides a potential mechanism for the observed body size-dependent ecosystem services of *P. natalis.*

*Pteropus natalis* foraging movements were negatively correlated to body mass both in terms of their magnitude and their frequency (Additional file 1: Table S7). These telemetry results are additionally supported by the results of a multi-year color band resighting program, which indicated that juveniles moved greater distances (45%) on average than adult conspecifics (C.M.T., pers., obs.). In the present study, larger *P. natalis* individuals visited fewer nightly foraging areas (Fig. 4D), traveled shorter distances from roost sites to nightly foraging areas (Fig. 4A), among nightly foraging areas (Fig. 4B and C) and across their foraging ranges (Fig. 3D), and had smaller foraging ranges (Fig. 3A) than smaller conspecifics (Additional file 1: Table S6) who covered a greater number of vegetation habitats. The observed links between foraging movements and body size found in this study are consistent with other studies of *Pteropus* species reporting smaller individuals traveling further distances and visiting a greater number of foraging areas than larger individuals [6, 55, 62, 63]. The dispersal of seeds and pollen plays a significant role in forest regeneration and maintenance, a key conservation issue among tropical forest ecosystems. The foraging movements of *P. natalis* demonstrates their ability to serve as a plant dispersal agent. Within a night, smaller *P. natalis* visited twice as many foraging areas as larger individuals (Fig. 4D) and therefore had greater potential to disperse pollen over longer distances.

Smaller *P. natalis* foraged on a greater diversity of plant species than their larger conspecifics (Fig. 5; Additional file 1: Table S7). On average the number of pollen types found on juvenile *P. natalis* was 64% more than the number of pollen types found on adults. Since bats groom themselves extensively while roosting, it is likely that few pollen grains should be retained from one night to the next [37]; therefore, the diversity of pollen collected from an individual is most likely indicative of the plant species visited during the night of capture. This study did not investigate the gut-passage time of seeds in *P. natalis*. However, the dispersal potential of pollen by *P. natalis* theoretically applies to the dispersal of seeds as the average gut-passage time for seeds is less than 30 min in a variety of frugivorous bat species [32, 59, 61, 78, 79, 82]. On Christmas Island the only native species, besides *P. natalis,* that serves as a disperser of both seeds and pollen is the much smaller Christmas Island white-eye (~ 14 g; *Zosterops natalis*). Although the movements of *Z. natalis* have not been studied, other *Zosterops* spp. typically have home ranges that are an order of magnitude smaller than those reported here for *P. natalis* (e.g., [1, 35], highlighting the critical nature of the long-distance pollen and seed dispersal services that particularly smaller *P. natalis* provide on Christmas Island.

Why are foraging movements body-size dependent in *P. natalis*? For many mammals body size is positively corelated with the size of foraging and home ranges [45, 54], as larger individuals tend to have a greater intrinsic capacity for movement [18, 66, 68] and need to cover more ground to meet their daily energy requirements [45, 54]. However, in our study larger individuals were in fact less mobile than smaller individuals overall; therefore, these explanations cannot account for the observed body size-dependent foraging movement dynamics of *P. natalis*. Our behavioral observations indicated that *P. natalis* engages in foraging resource defense through a variety of agonistic behaviors, including vocalizations, wing spreading, chasing, fighting, and scent marking as similarly reported among other *Pteropus* species [6, 14, 60, 71, 81, 89, 90]. Resource defense generally functions to provide a dominant individual with an adequate supply of some critical resource (often food) at the expense of less dominant individuals (usually conspecifics; [16, 44] who are forced to move more often to acquire undefended foraging resources [16, 28]. Although not directly tested in our study, in many territorial species, dominance is linked to body size [21, 22, 38, 52, 87], and the observed body size-dependent foraging movements of *P. natalis* are consistent with those of other nectivorous animals, including bats, that actively defend foraging resources [44, 46].

It could be argued that age rather than body size explains the differences in foraging movements and pollen diversity, as body size and age are related and, in particular, volant juvenile flying-foxes may travel on long, so called ‘exploratory flights’ (e.g., [6]. However, while age is likely a contributing factor to differences in movements at the population level, it cannot explain the differences in movements observed in this study: tracked individuals were adults with the exception of one sub-adult. In addition, the number of pollen types found on caught individuals was negatively correlated to body mass within age class, including within the subset of tracked individuals for which pollen counts were available, clearly showing an effect of body mass on pollen diversity irrespective of age. While anecdotal observations suggest that smaller flying-fox individuals can be displaced from foraging sites by larger, dominant individuals (e.g., [53], to our knowledge, no studies of free ranging populations have directly tested whether foraging resource defense is body size-dependent in this taxon. This is likely due to the difficulty of observing individuals of known body mass interacting at foraging sites; however, diet and behavioral studies on captive populations of *P. livingstonii* demonstrated that older individuals [22], were more dominant and defended and/or displace smaller individuals from foraging resources [52, 87].

Alternatively, *P. natalis’* size-dependent foraging movements could be the result of producer-scrounger dynamics (e.g., [7, 36]. For example, larger-sized ‘scroungers’ could ‘eavesdrop’ on the foraging success of smaller ‘producers’ to help them locate and then usurp foraging resources, forcing the smaller producers to move to another foraging location. However, similarly to other *Pteropus* studies [26, 50, 84], we found that tracked individuals of all sizes regularly returned to the same foraging locations night after night, implying that individuals base their foraging decisions at least in part on their prior knowledge of the locations of foraging resources. Therefore, producer-scrounger dynamics do not present a mutually-exclusive alternative to resource defense as an explanation for the body size-dependent foraging movements observed in this species. Research involving systematic behavioral observations on individuals of known body mass interacting while foraging in the wild is needed to further investigate the social dynamics of *P. natalis* and to better understand how body size-dependent social interactions affect foraging movements and their consequences for flying-fox ecosystem services.

Our findings indicate that smaller *P. natalis* are the primary contributors to long distance dispersal of pollen and seeds provided by this species. Similar results were found in populations of *P. conspicillatus*, where ‘residents’, appeared to provide reduced dispersal distances compared to transient ‘raiders’ [71]. Territorial behaviors are primarily a function of competition, which can be dependent on population densities [15, 48]. A number of studies, including those conducted on *Pteropus* species, have shown that decreasing population densities of seed dispersers and pollinators can disrupt dispersal mutualisms by reducing the quantity of propagules dispersed or the distances over which they are transported [53, 83, 92]. Reduced competitive interactions at foraging sites can affect such dispersal mutualisms in a nonlinear way, so that flying-foxes may cease to be effective plant vectors long before becoming rare [53]. Therefore, the current threated status of *P. natalis* on Christmas Island is of concern as the effectiveness of the species as a vector of pollen and seeds may rely on the synergistic effects between competitive interactions and decreasing density, and hence population.

## Conclusion

The spatial movements of *P. natalis*, which varied considerably among individuals and were significantly linked to body mass, have important implications for the reproductive biology of a large number of Christmas Island’s plant species. Animal dispersers are crucial to facilitate and maintain gene flow among many plant populations [73]. Many of Christmas Island’s native plant species are “Chiropterophilous” and almost all are self-incompatible hermaphrodites that require cross-pollination to set fruit [65]. Therefore, the direct ecological impacts caused by the further decline or loss of *P. natalis* could be severe and may in turn precipitate indirect negative impacts to other species throughout Christmas Island’s unique ecological communities. In addition, the maintenance of population diversity contributes to the long-term conservation of essential ecosystem processes [74], and the findings of our study imply that changes to the demographic composition of *P. natalis* could exacerbate the ecological impacts of the species’ decline on Christmas Island. We therefore recommend that the disparate ecosystem services provided by the demographic diversity should be considered and incorporated more explicitly into studies and management actions of this and other insular *Pteropus* species, for a more holistic approach benefitting both flying-foxes and the ecosystems they service.

## Supplementary Information

**Additioanal file 1: Table S1**. Known and suspected food plants for Christmas Island flying-foxes (*Pteropus natalis)*. **Table S2**. Classification of habitat types on Christmas Island. **Figure S1**. The proportions and capture times for pollen samples collected from Christmas Island flying-foxes (*Pteropus natalis*). **Table S3**. Christmas Island flying-foxes (*Pteropus natalis*) fitted and tracked with GPS telemetry nodes. **Table S4**. Overlap in foraging metrics of simultaneously tracked Christmas Island flying-foxes (*Pteropus natalis*). **Table S5**. Akaike information criterion (AIC_c_) model selection for foraging movements by the Christmas Island flying-fox (*Pteropus natalis*). **Table S6**. Foraging range movements of 24 Christmas Island flying-foxes (*Pteropus natalis*). **Figure S2**. Location data identifying movement patterns for all Christmas Island flying-foxes (*Pteropus natalis*) fit with GPS telemetry nodes between August 2015 and November 2017. **Table S7**. Results of generalized linear models predicting foraging movements of Christmas Island flying-foxes (*Pteropus natalis*).

**Additioanal file 2.** Detailed results on observations of foraging resource defense by Christmas Island flying-foxes (*Pteropus natalis*).

## Data Availability

The GPS data supporting the conclusion of this research are available on movebank.org; study name: “Movements of the Christmas Island flying fox, Australia”.

## Abbreviations

MBKE: Movement-based kernel estimates
UD: Utilization distributions
FR: Foraging range
FA: Foraging area
LAX: Long axis across FR
GLM: General linear models
LMM: Linear mixed-effects model
GLMM: Generalized linear mixed-effects model
PCA: Principal component analysis
AIC: Akaike information criterion
