# Supplementary material for: Body-size dependent foraging strategies in the Christmas Island flying-fox: implications for seed and pollen dispersal within a threatened island ecosystem
Source: Mov Ecol. 2022 Apr 11;10:19. doi: 10.1186/s40462-022-00315-8 (PMC8996557; doi:10.1186/s40462-022-00315-8)
Supplement: Supplementary file 1 — Additioanal file 1: Table S1. Known and suspected food plants for Christmas Island flying-foxes (Pteropus natalis). Table S2. Classification of habitat types on Christmas Island. Figure S1. The proportions and capture times for pollen samples collected from Christmas Island flying-foxes (Pteropus natalis). Table S3. Christmas Island flying-foxes (Pteropus natalis) fitted and tracked with GPS telemetry nodes. Table S4. Overlap in foraging metrics of simultaneously tracked Christmas Island flying-foxes (Pteropus natalis). Table S5. Akaike information criterion (AICc) model selection for foraging movements by the Christmas Island flying-fox (Pteropus natalis). Table S6. Foraging range movements of 24 Christmas Island flying-foxes (Pteropus natalis). Figure S2. Location data identifying movement patterns for all Christmas Island flying-foxes (Pteropus natalis) fit with GPS telemetry nodes between August 2015 and November 2017. Table S7. Results of generalized linear models predicting foraging movements of Christmas Island flying-foxes (Pteropus natalis). [file 40462_2022_315_MOESM1_ESM.pdf]

Additional File 1: Supplemental tables and figures

Table S1. Known and suspected food plants used by Christmas Island flying-foxes (*Pteropus natalis*). Species denoted with (*) indicate an observation from another study. Species denoted with (†) indicate unconfirmed food plants of *P. natalis* considered as highly likely as they appear on Christmas Island and are reported as a food resource in the diet of other *Pteropus* spp.

Family	Species	Fruits	Flowers	Leaves	Stems	Source
**Native**						
Anacardiaceae	*Spondias cytherea†*	+	+			9
Arecaceae	*Arenga listeri*		+			1,3,5
Boraginaceae	*Cordia subcordata*		+			1
Calophyllaceae	*Calophyllum inophyllum*	+		+†		1, †12
Cannabaceae	*Celtis timorensis*	+				3,5,6
Combretaceae	*Terminalia catappa*	+	+			1,3,5,6
Euphorbiaceae	*Macaranga tanarius*		+		+	1,3,5,6
Fabaceae	*Erythrina variegata*		+			1
Fabaceae	*Inocarpus fagifer*	+	+	+		1,2,5
Hernandiaceae	*Hernandia ovigera†*		+			10
Lecythidaceae	*Barringtonia racemosa*		+			1,3,5
Meliaceae	*Dysoxylum gaudichaudianum*		+			1,3,5
Moraceae	*Ficus microcarpa*	+		+*		1,3,5, *3
Moraceae	*Ficus saxophila*	+		+*		1,3,*3
Moraceae	*Maclura cochinchinensis*		+			6
Myrtaceae	*Syzigium nervosum*	+	+			1,3,5,12
Pandanaceae	*Pandanas chrismatensis*		+			1
Pandanaceae	*Pandanus elatus†*	+	+			9
Pittosporaceae	*Pittosporum ferrugineum*	+				1
Rhizophoraceae	*Bruguiera gymnorrhiza*		+			1
Rubiaceae	*Guettarda speciosa*		+	+	+	1
Rubiaceae	*Morinda citrifolia*	+*	+			3,*7
Sapindaceae	*Allophylus cobbe†*		+			11
Sapindaceae	*Tristiropsis acutangula*	+	+			3,5,6
Sapotaceae	*Planchonella nitida*	+	+			1,2,3,5

Sapotaceae	*Planchonella nitida*†			+	12
Urticaceae	*Dendrocnide sp.*		+		1,3,5
Urticaceae	*Pipturus argenteus*	+	+*		1, *(3,5)

Introduced

Anacardiaceae	*Anacardium occidentale*	+			6
Anacardiaceae	*Mangifera indica*	+			1,3,5,6
Anacardiaceae	*Mangifera odorata*	+			1,3,5,6
Annonaceae	*Annona muricata*	+			1,5,6
Annonaceae	*Annona reticulata*	+			6
Araliaceae	*Schefflera actinophylla*	+	+		1,6
Arecaceae	*Cocos nucifera*		+		1,3,5,6
Bignoniaceae	*Spathodea campanulata*†		+		8
Caricaceae	*Carica papaya*	+			1,2,3,5,6
Clusiaceae	*Garcinia mangostana*	+			7
Clusiaceae	*Garcinia xanthochymus*	+			7
Ebenaceae	*Diospyros digyna*	+			6
Euphorbiaceae	*Hevea brasiliensis*		+		1
Euphorbiaceae	*Manihot glaziovii*		+		1,6
Euphorbiacea	*spp. Type 1*		+		1
Euphorbiacea	*Spp. Type 2*		+		1
Lauraceae	*Persea Americana*	+	+		1,6
Lecythidaceae	*Barringtonia asiatica*		+		3,6
Lythraceae	*Punica granatum*†	+	+		8
Malvaceae	*Theobroma cacao*	+			1
Meliaceae	*Melia azedarach*		+		3,5
Moraceae	*Atrocarpus altilis*	+			1
Moraceae	*Atrocarpus heterophyllus*	+			1,6
Muntingiaceae	*Muntingia calabura*	+	+		1,3,4,5,6
Musaceae	*Musa spp.*	+	+		1,2
Myrtaceae	*Psidium guajava*	+*	+		1, *(3,5,6)
Myrtaceae	*Syzigium jambos*	+	+		1,12
Passifloraceae	*Passiflora foetida var. hispida*	+*	+		1, *(7)
Rutaceae	*Citrus maxima*		+		6
Sapindaceae	*Nephelium lappaceum*	+			7
Sapotaceae	*Manilkara zapota*	+	+		1,5,6
Solanaceae	*Physalis sp.*		+		3,5

Source: 1 = This study, 2 = Andrews 1900, 3 = Tidemann 1985, 4 = Corbett et al. 2003, 5 = Orchard et al. 2006, 6 = James 2007, 7 = Christmas Island National Parks (pers., com.), 8 = Marshal 1985, 9 = Banack 1998, 10 = Wiles and Fujita 1992, 11 = Picot 2007, 12 = Nelson 2000.

Table S2. Classification of habitat types on Christmas Island.

Habitat type		Vegetation height categories	Description	Indicator species	Area (ha)
**Evergreen forest**	**Closed canopy evergreen forest (tall - moderate)**	≤ 5 - 40 m	Generally found on the plateau and terraces, with a closed uneven canopy up to 40 m in height. Some trees emerge up to 10 m above the canopy. Often supports ferns and orchids, young palms and lilies in the understory.	*Bolbitis hetroclita, Syzigium nervosum, Hernandia ovigera, Planchonella nitida, Pisonia umbellifera, Corymborkis veratrifolia, Ehretia javanica*	6536.96
**Semi-deciduous**	**Semi-deciduous forest**	10 - 20 m	Generally found on the slopes and terraces down to the coast - and some plateau areas. Higher occurrence of semi-deciduous trees compared to Closed Canopy Evergreen, which lose a portion of leaves during the dry season. Tree height generally 10-25m	*Terminalia, Gyrocarpus, Erythrina variegate, Premna seratafolia, Pisonia grandis, Ochrosia ackeringae*	1977.10
	**Semi-deciduous scrub**	≤ 5 -10 m	Found on the terraces, steep slopes and inland cliffs. Semi-deciduous canopy with vines and shrub understory Tree height generally <10m.	*Colubrina pendunculata, Canavalia cathartica, Carmona retusa, Cycads,*	1343.58
**Perennial wetland forest**	***Inocarpus fagifer* dominant**	≤ 5 - 30 m	Areas of fresh water runoff on the lower terraces dominated by Inocarpus fagifer.	*Inocarpus fagifer*	34.47
	***Hibiscus tiliaceus* dominant**	≤ 5 - 20 m	Areas of fresh water runoff on the shore terrace dominated by *Hibiscus tiliaceus.*	*Hibiscus tiliaceus*	45.10
	***Bruguiera* dominant**	≤ 5 - 25 m	A single patch of vegetation dominated by *Bruguiera gymnorhiza* at Hosnie Springs. Occurring in an area of fresh water runoff on the shore terrace.	*Bruguiera gymnorhiza*	1.70

**Coastal fringe**	**Coastal herbland**	≤ 5 - 10 m	Found between the coastal scrub and coastal cliffs in exposed areas. Class is dominated by low-lying herbs, sedges and grasses.	*Portulaca tuberosa, Ishaemum nativitalis, Oplismenus composits, Sporobolus virganicus*	9.28
	**Coastal shrubland**	≤ 5 - 20 m	Dense salt-tolerant vegetation growing between the coastal herb land and the terrace cliffs.	*Pandanus christmatensis, Scavola, Pemphis, Argusia argentia, Cordia cordata, Guetarda*	176.25
**Rehabiliation**	**Rehabilitation**	≤ 5 - 35 m	Areas where forest rehabilitation has taken place. The standard of forest varies depending on the type of rehabilitation completed, species planted and management regime.	A mix of up to 30 native tree species when initially planted, dependent on characteristic of the site and year of rehabilitation. *Macaranga, Dysoxylum, Callophylum, Tristeropsis*	251.81
**Regrowth**	**Regrowth**	≤ 5 - 35 m	Generally well developed regrowth vegetation over 5m mean tree height. May include some introduced or weed species.	Various species – dependent on adjacent vegetation	1432.32
**Weed dominated and pioneer growth**	***Leucaena leucocephala***	≤ 5 - 15 m	Monoculture of *Leucaena leucocephala.* Often occurring as regrowth in previously cleared areas.	*Leucaena leucocephala*	90.87
	**Fern field**	≤ 5 - 15 m	Expanse of low-lying ferns often growing on limestone pinnacles.	*Nephrolepis bisserata, Microsorum scolopendria, Psilotum nudum*	407.18
	**Mixed weed and pioneer regrowth**	< 5 m	Regrowth vegetation with a mean tree height of <5m. Can vary between native and introduced species depending on the location and time since clearing. Tends to have a higher occurrence of weed species compared to the 'Regrowth' category.	*Muntingia calabura, Psidium sp.(Guava), Mimosa, Passionfruit, Macaranga*	517.32

**Not vegetated**	**Bare ground**	Roads, airport, sporting, waste disposal	215.67
	**Residential**		81.08
	**Infrastructure**		232.40
	**Mining**		122.15
	**Coastal pinnacles / sand**		117.77

Figure S1. The proportions and capture times for pollen samples collected from Christmas Island flying-foxes (*Pteropus natalis*). From the total sample obtained (n =216), 115 pollen samples (—) were used to assess foraging resource use. One hundred and one (- - -) were removed because they did not contain any pollen (*n* = 46) or because they contained a pollen load < 20 pollen grains (n = 55).

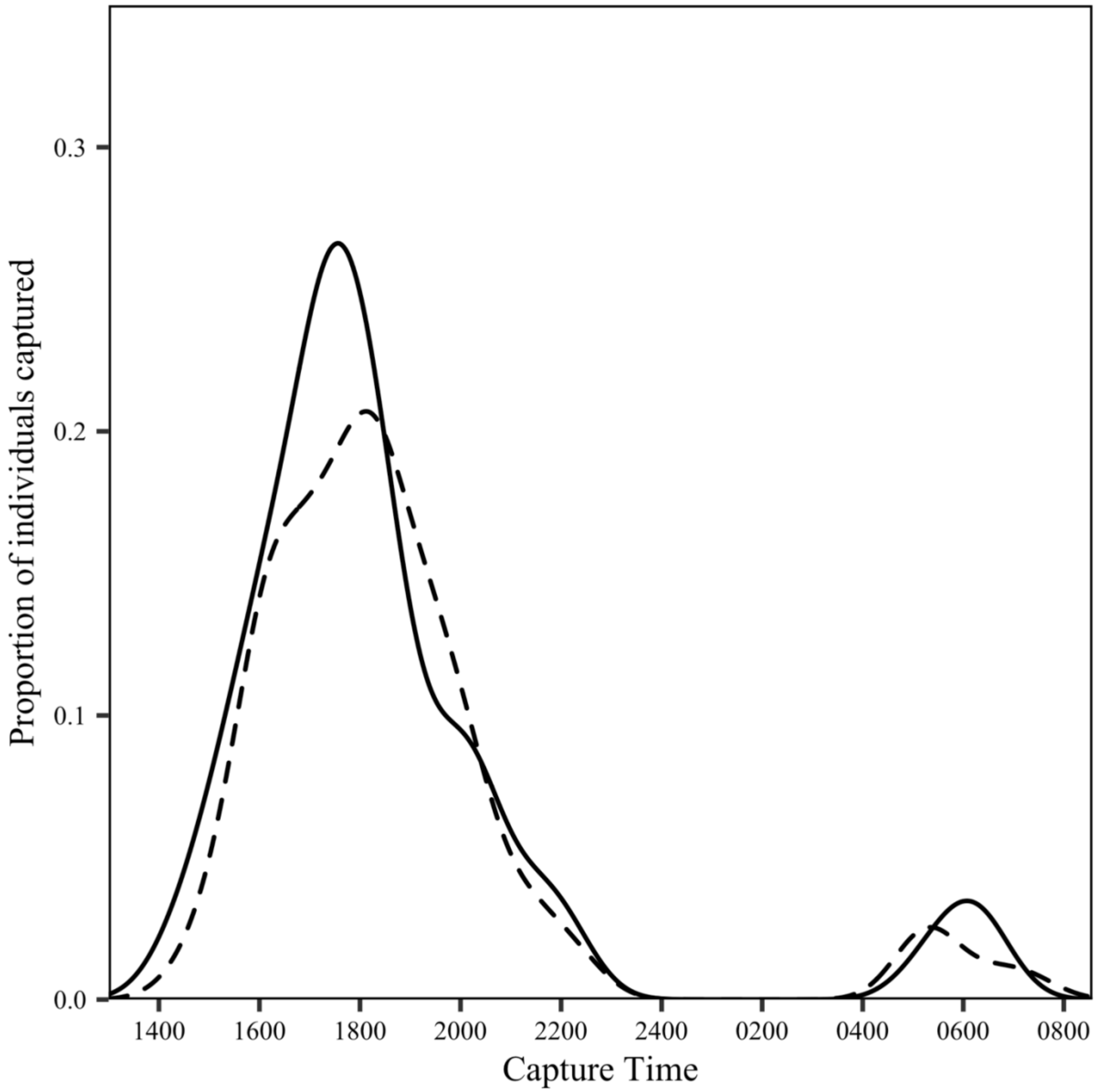

Table S3. Deployment date, deployment location, total number of GPS fixes (*n*) and nights tracked, number of 20 minute GPS fixes (*n*) and nights tracked, and number of 20 and 60 minute GPS fixes (*n*) and nights tracked, sex and age class, body mass and skeletal size (derived from a Principle component analysis [PCA] on the measurements of forearm length, thumb length, claw length and tibia length) for Christmas Island flying-foxes (*Pteropus natalis*) fitted and tracked with GPS telemetry nodes between August 2015 and November 2017.

Tag ID	Deployment date	Deployment location	Total GPS fixes (*n*) and nights tracked	20 min GPS fixes (*n*) and nights tracked	20 and 60 min GPS fixes (*n*) and nights tracked	Sex/age class	Body mass (g)	Skeletal size (PCA Score)
730	10-Aug-15	Territory Day Park	(310); 16	(299); 10	(299); 10	♂ Adult	436	0.41
602	10-Aug-15	Territory Day Park	(219); 11	(130); 4	(151); 7	♂ Adult	414	0.26
261	12-Aug-15	Territory Day Park	(284); 15	(157); 5	(208); 10	♀ Adult	413	-5.02
729	7-Dec-15	Hosnies Spring	(152); 6	(141); 4	(141); 4	♂ Adult	414	0.37
577	4-Jan-16	Pink House Research Station	(321); 13	(272); 8	(283); 7	♂ Adult	394	1.36
423	25-Jan-16	Pink House Research Station	(260); 11	(156); 5	(228); 9	♂ Adult	395	0.84
671	27-Jan-16	Territory Day Park	(125); 4	(116); 3	(125); 4	♂ Adult	418	-0.85
556	10-Sep-16	Territory Day Park	(528); 11	(528); 11	(528); 11	♂ Adult	505	2.57
1224	15-Sep-16	Settlement	(98); 3	(87); 3	(87); 3	♂ Sub-adult	413	-0.37
1041	1-Oct-16	Flying Fish Cove	(246); 11	(227); 6	(227); 6	♂ Adult	395	-0.82
1223	1-Oct-16	Settlement	(147); 5	(134); 5	(141); 4	♂ Adult	515	1.52
812	22-May-17	Lily Beach Rd	(157); 5	(157); 5	(157); 5	♂ Adult	478	-0.21
797	27-Sep-17	Grants Well	(146); 7	(98); 3	(134); 6	♀ Adult	414	-2.90
806	23-Oct-17	Hughes Dale	(399); 19	(246); 6	(354); 15	♂ Adult	407	0.66
777	23-Oct-17	Hughes Dale	(381); 28	(219); 6	(336); 15	♀ Adult	459	1.21
840	26-Oct-17	Hughes Dale	(378); 24	(343); 9	(343); 9	♀ Adult	448	-0.63
486	26-Oct-17	Hughes Dale	(131); 4	(121); 4	(121); 4	♀ Adult	416	-1.12

776	13-Nov-17	Hosnies Spring	(170); 4	(128); 3	(128); 3	♂ Adult	374	-0.21
434	22-Nov-17	McMicken Point	(197); 5	(177); 5	(177); 5	♂ Adult	392	0.78
738	17-Aug-16	McMicken Point	(321); 13	(194); 6	(248); 10	♂ Adult	406	1.41
570	27-Jul-16	Flying Fish Cove	(310); 13	(184); 5	(234); 9	♀ Adult	423	-0.85
733	25-Jan-16	Flying Fish Cove	(118); 3	(103); 3	(103); 3	♂ Adult	406	2.32
606	27-Jul-16	Flying Fish Cove	(285); 12	(205); 6	(250); 9	♀ Adult	466	0.34
737	29-Jul-16	Flying Fish Cove	(248); 10	(194); 5	(221); 7	♀ Adult	432	-1.06

Table S4. Percent overlap of foraging areas (FA, top right diagonal) and the distance (median; interquartile range [IQR]) between foraging locations (grey, bottom left) for three separate groups of Christmas Island flying-foxes (*Pteropus natalis*) that were simultaneously tracked in August 2015 (adult male 730, adult female 261, and adult male 602), July – August 2016 (adult female 737, adult female 570, and adult female 606) and October – November 2017 (adult female 486, adult female 840, adult female 777, and adult male 806). For percent overlap of FA (top right diagonal), *Denotes values for overlap percentages for individuals on the left side of the matrix and a (†) denotes overlap percentages for individuals on the top of the matrix.

Location: Flying fish cove (12 - 25 August 2015)			
GPS node † *	730	261	602
730	- -	12.22* 2.24†	2.22* 2.11†
261	172.08; (124.91 - 174.53)	- -	3.26* 16.84†
602	70.34; (43.71 - 82.22)	70.21; (64.86 - 505.77)	- -

Location: Flying fish cove (27 July - 5 August 2016)			
GPS node † *	737	570	606
737	- -	35.0*	58.33*

		18.92†	22.87†
570	169.23; (75.25 - 326.89)	- -	9.91* 7.19†
606	24.28; (15.43 - 34.70)	78.72; (49.08 - 284.40)	- -

Location: Hughs Dale (26-29 October 2017)

GPS node † *	486	840	777	806
486	- -	15.09* 0.96†	3.77* 2.63†	37.74* 2.46†
840	147.56; (44.25 - 231.08)	- -	0* 0†	13.88* 14.28†
777	50.09; (26.82 - 59.61)	No overlap	- -	13.15* 1.23†
806	405.41; (205.21 - 423.99)	57.86; (33.70 - 119.89)	80.10; (41.21 - 98.26)	- -

30

Table S5. Aikake Information Criterion (AIC$_c$), delta scores, weights and regression coefficients of the explanatory variables for the five best models for foraging range (FR), foraging area (FA), long axis across the foraging range (LAX), the number of pollen types, the number of vegetation types in the FR and FA, nightly dispersal, distance from roost to nightly FAs, distance between nightly used FAs, and the number of nightly used FAs for Christmas Island flying-foxes (*Pteropus natalis*). Categorical fixed effects get a + when they are included in the model. Only explanatory variables used in the models are provided in the table. The explanatory variables with interactions for body mass and sex, skeletal size and sex, body mass and time of year were included in the analysis but left out of the table as they were not included in any of the five best models or in models with $\Delta AIC_c \leq 2$. Models with $\Delta AIC_c \leq 2$ used in model averaging are marked with an asterisks.

Response variable	Intercept	Body Mass	Skeletal size	Sex	Age Class	Time of year	Rain	Number of GPS points	Body mass* Rain	Body mass* Age class	Time of Capture	df	AIC$_c$	ΔAIC$_c$	Weight
Generalized linear models															
	-0.67	-0.74					-0.45	0.00				5	62.60	0.00*	0.31
	0.00	-0.60					-0.35					4	63.20	0.58*	0.23
Foraging Range (FR)	0.04	-0.59					-0.19		0.35			5	63.70	1.08*	0.18
	-0.52	-0.71					-0.32	0.00	0.26			6	64.70	2.05	0.11
	-0.74	-0.71	-0.10				-0.43	0.00				6	65.10	2.44	0.90
	-1.03	-0.64					-0.34	0.01				5	68.20	0.00*	0.31
Foraging Area (FA)	-0.78	-0.55						0.00				4	68.70	0.52*	0.24
	0.00	-0.40										3	69.80	1.60*	0.14

	-0.92	-0.52	-0.15					0.00				5	70.20	2.00*	0.12
	-1.12	-0.60	-0.12				-0.32	0.01				6	70.40	2.22	0.11
Long-axis (LAX)	-0.74	-0.56					-0.53	0.00				5	65.80	0.00*	0.32
	0.00	-0.46					-0.44					4	66.30	0.50*	0.25
	0.05	-0.44					-0.27		0.36			5	66.90	1.15*	0.18
	-0.57	-0.53					-0.40	0.00	0.24			6	68.20	2.46	0.09
	-0.79	-0.53	-0.09				-0.50	0.00				6	68.20	2.46	0.09
Number of Pollen Types	4.68	-0.01		+	+		-0.01					6	425.20	0.00*	0.13
	4.41	-0.01			+		-0.01					5	425.70	0.54*	0.10
	4.74	-0.01		+	+							5	427.10	1.92*	0.51
	3.73	-0.01		+	+		-0.01			+		8	427.10	1.93*	0.51
	4.73	-0.01		+	+		-1.01				0.00	7	427.30	2.04	0.05
No. Vegetation Habitats (FR)	-0.68	-0.74					-0.44	0.00				5	63.00	0.00*	0.32
	0.00	-0.60					-0.33					4	63.70	0.63*	0.24
	0.04	-0.59					-0.18		0.32			5	64.60	1.58*	0.15
	0.00	-0.56										3	65.00	1.94*	0.12
	-0.55	-0.71					-0.31	0.00	0.23			6	65.50	2.42	0.10
No. Vegetation Habitats (FA)	-0.76	-0.58						0.00				4	67.60	0.00*	0.39
	0.00	-0.44										3	68.70	1.06*	0.23
	-0.85	-0.56	-0.10					0.00				5	70.10	2.44	0.11
	-0.86	-0.62					-0.14	0.00				5	70.20	2.58	0.11
	-0.96	-0.59				0.03		0.00				5	70.50	2.89	0.09
Linear mixed effects models															
	0.10	-0.33										4	530.00	0.00*	0.49

	0.11						3	531.08	1.85*	0.19
Nightly dispersal	0.01	-0.32		+			5	532.50	2.51	0.14
	-0.37		-0.25	+			5	533.00	3.02	0.11
	-0.06			+			4	533.70	3.72	0.08
Distance: Roost to FAs	-0.01	-0.30					4	1320.90	0.00*	0.55
	-0.05						3	1323.60	2.78	0.14
	-0.08	-0.29		+			5	1323.70	2.83	0.14
	-0.50		-0.24	+			5	1324.20	3.31	0.11
	-0.01	-0.27	-0.08				5	1325.10	4.22	0.07
Distance: Between FAs	0.01	-0.31					4	1038.60	0.00*	0.58
	0.01	-0.31			-0.09		5	1041.50	2.92	0.13
	0.05	-0.31		+			5	1041.60	2.97	0.13
	-0.60						3	1042.20	3.58	0.10
	0.01	-0.28	-0.08				5	1042.90	4.36	0.07
<u>Generalized linear mixed effects models</u>										
No. Nightly FAs	1.11	-0.35					3	394.50	0.00*	0.34
	1.27	-0.34				-0.02	4	395.20	0.68*	0.24
	1.11	-0.34	-0.04				4	395.80	1.23*	0.18

	1.11	-0.35		-0.01	4	396.60	2.07	0.12
	1.10	-0.34	+		4	396.70	2.13	0.12

Table S6. Foraging range (FR, 0.95 kernel), foraging areas (FA, 0.30 kernel), distance traveled (median and interquartile range [IQR]) from roost site to each nightly used (FAs), distance traveled (median and interquartile range [IQR]), between nightly used (FAs), nightly dispersal (median and interquartile range [IQR]), number of nightly and total foraging sites (median, range and total), and long axis across the foraging range (LAX) for 24 Christmas Island flying-foxes (*Pteropus natalis*) tracked with GPS telemetry nodes between August 2015 and November 2017. For visualization of movements for all individuals please refer to supplementary material 1: Figure S2.

Tag ID	Sex/age class	Body mass (g)	FR 95% Kernel (ha)	FA 30% Kernel (ha)	Distance (m) from roost to foraging area (FA); IQR	Distance (m) between foraging areas (FA); IQR	Nightly Dispersal (km); IQR	No. nightly and total foraging sites	Long Axis (km)
730	♂ Adult	436	40.45	0.9	62.16; (15.59 - 91.35)	76.06; (75.11 - 616.86)	0.69; (0.24 - 0.71)	3 (1 - 3); 4	3.64
602	♂ Adult	414	32.09	0.95	435.68; (61.42 - 707.78)	435.67; (286.43 - 723.04)	0.81; (0.60 - 0.83)	3 (2 - 3); 6	0.98
261	♀ Adult	413	388.38	4.9	330.64; (128.56 - 745.65)	297.34; (165.20 - 542.91)	0.79; (0.41 - 0.56)	5 (3 - 7); 16	6.05
729	♂ Adult	414	463.84	14.77	1,614.75; (48.80 - 2,060.71)	1,679.62; (207.68 - 2,583.04)	2.36; (2.20 - 4.01)	4 (2 - 5); 7	5.05
577	♂ Adult	394	221.33	8	61.89; (31.45 - 749.15)	148.39; (46.44 - 304.80)	0.30; (0.09 - 0.78)	2 (1 - 3); 5	5.29

423	♂ Adult	395	300.3	2.69	325.74; (109.21 - 438.44)	165.67; (55.32 - 460.61)	0.59; (0.48 - 1.56)	5 (2 - 7); 8	3.48
671	♂ Adult	418	573.69	4.7	3,239.38; (1,190.22 - 4,711.44)	1,712.72; (276.36 - 2,275.22)	3.47; (1.87 - 4.62)	1 (1 - 5); 5	5.09
556	♂ Adult	505	19.36	1.02	32.33; (20.58 - 615.06)	120.42; (77.44 - 614.95)	0.18; (0.15 - 0.69)	1 (1 - 4); 4	1.07
1224	♂ Sub-adult	413	25.67	0.3	122.33; (44.41 - 247.31)	126.55; (111.76 - 510.69)	0.65; (0.42 - 1.60)	2 (2 - 3); 5	2.55
1041	♂ Adult	395	5.9	0.53	177.49; (73.92 - 192.78)	104.20; (68.87 - 166.02)	0.24; (0.19 - 0.25)	4 (3 - 6); 8	0.25
1223	♂ Adult	515	4.33	0.1	121.06; (71.02 - 129.21)	109.68; (59.98 - 173.34)	0.25; (0.21 - 0.31)	3 (1 - 3); 6	0.46
812	♂ Adult	478	12.33	1.01	101.07; (83.68 - 150.75)	124.46; (66.36 - 172.52)	0.42; (0.33 - 0.49)	3 (1 - 4); 7	0.63
797	♀ Adult	414	488.14	8.51	886.03; (148.74 - 1,422.85)	1,017.83; (454.39 - 1,692.36)	1.94; (1.13 - 2.83)	4 (2 - 4); 11	6.75
806	♂ Adult	407	329.63	8.12	310.03; (133.82 - 1,469.63)	275.44; (165.46 - 413.01)	0.56; (0.43 - 1.66)	5 (2 - 8); 14	3.66
777	♀ Adult	459	10.64	0.76	48.6; (28.15 - 64.10)	95.49; (81.05 - 110.34)	0.13; (0.12 - 0.14)	2 (1 - 2); 3	0.49
840	♀ Adult	448	157.98	8.36	220.82; (133.85 - 607.35)	196.44; (136.27 - 508.95)	0.66; (0.40 - 1.00)	3 (2 - 5); 11	3.12

486	♀ Adult	416	13.57	0.53	57.01; (38.88 - 107.94)	93.77; (68.77 - 136.39)	0.28; (0.21 - 0.36)	3 (2 - 4); 7	0.54
776	♂ Adult	374	1935.57	65.97	3,875.09; (2,260.26 - 4,952.40)	1,282.54; (303.11 - 3,769.06)	5.01; (5.01 - 6.95)	7 (1 - 9); 9	8.89
434	♂ Adult	392	131.81	0.54	694.44; (639.80 - 825.95)	265.13; (99.47 - 607.67)	0.90; (0.83 - 0.95)	5 (2 - 8); 17	3.1
738	♂ Adult	406	510.97	54.62	813.69; (392.32 - 1,526.94)	813.32; (535.59 - 1,234.46)	2.04; (1.46 - 2.25)	4 (1 - 5); 11	6.11
570	♀ Adult	423	19.54	1.53	387.39; (50.35 - 437.74)	281.83; (61.83 - 377.71)	0.60; (0.45 - 0.71)	4 (1 - 5); 8	0.92
733	♂ Adult	406	6.17	0.23	782.01; (19.11 - 328.46)	239.90; (186.87 - 277.52)	0.39; (0.26 - 0.44)	3 (1 - 4); 5	0.44
606	♀ Adult	466	12.29	0.6	26.73; (15.97 - 506.59)	506.02; (101.72 - 510.89)	0.52; (0.24 - 0.57)	2 (1 - 2); 4	0.83
737	♀ Adult	432	14.61	1.11	300.41; (291.85 - 341.83)	316.67; (300.72 - 634.76)	0.67; (0.52 - 0.68)	3 (2 - 3); 3	0.72

Figure S2. Location data identifying movement patterns for all Christmas Island flying-foxes (*Pteropus natalis*) fit with GPS telemetry nodes between August 2015 and November 2017.

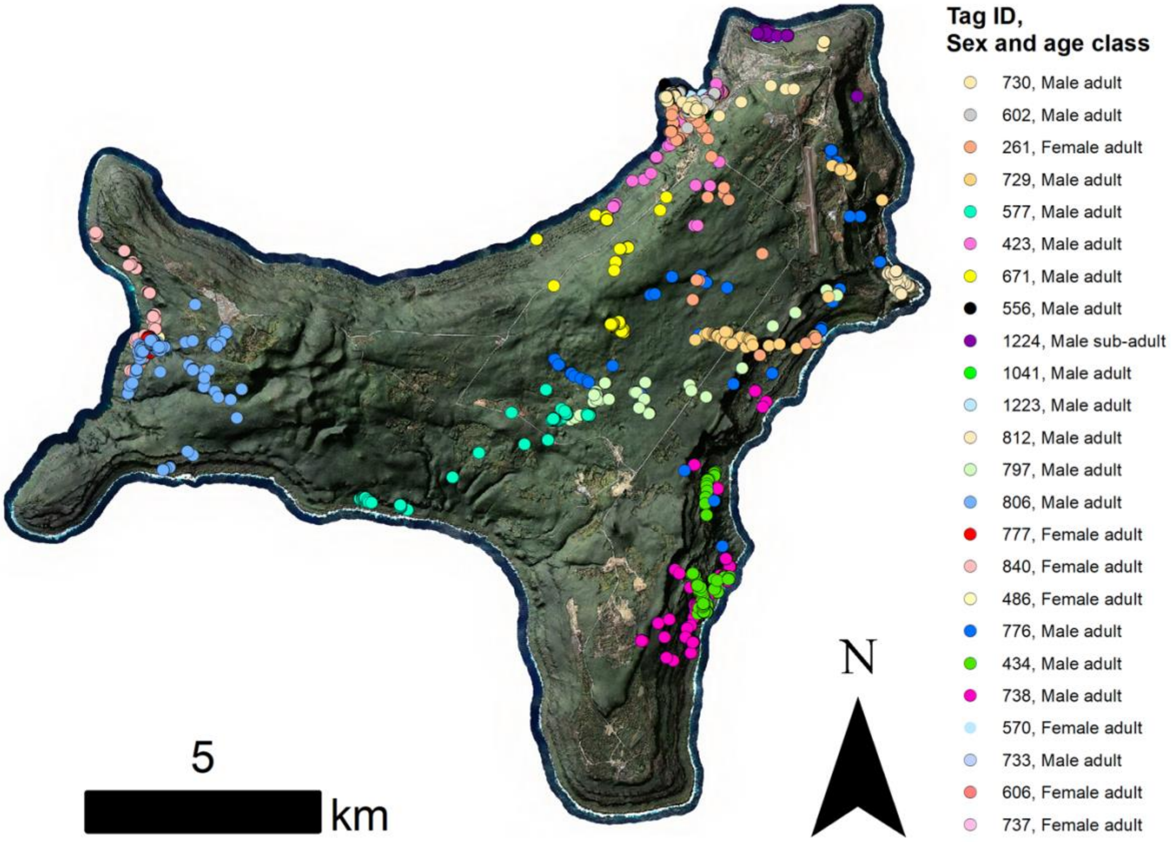

Table S7. Results of the best general linear models (GLM), according to Akaike information criterion AIC_c, that predicted changes in the size of foraging range (FR), foraging area (FA), long axis across the foraging range (LAX), number of pollen types, number of vegetation habitats in the FR and FA, nightly dispersal, distance from roost to nightly FAs, distance between nightly used FAs, and the number of nightly used FAs for Christmas Island flying-foxes (*Pteropus natalis*) captured between august 2015 and November 2017.

Response variable	Explanatory variables	Coef. β	*SE*	*z*-value	*P*-value	*R2*
Generalized linear models						
Foraging Range (FR)	Intercept	-0.274	0.441	0.607	0.544	
	Body mass	-0.657	0.182	3.427	***0.001***	46.19%
	Rain	-0.356	0.202	1.687	0.092	11.18%
	Number of GPS points	0.001	0.002	0.690	0.490	10.39%
	Body mass*rain	0.087	0.189	0.453	0.650	6.28%
Foraging Area (FA)	Intercept	-0.760	0.545	1.347	0.178	
	Body mass	-0.554	0.213	2.466	***0.014***	33.31%
	Rain	-0.131	0.202	0.638	0.524	13.17%
	Number of GPS points	0.004	0.003	1.428	0.153	28.15%
	Skeletal size	-0.021	0.068	0.297	0.766	5.63%
Long-axis (LAX)	Intercept	-0.306	0.489	0.609	0.543	
	Body mass	-0.501	0.183	2.594	***0.009***	33.15%
	Rain	-0.437	0.207	2.010	***0.044***	15.49%
	Number of GPS points	0.001	0.002	0.685	0.494	10.47%
	Body mass*rain	0.087	0.195	0.437	0.662	5.79%
No. of Pollen Types	Intercept	4.464	0.862	5.132	0.000	
	Age class	-0.649	0.967	0.668	0.504	7.97%
	Body mass	-0.008	0.002	3.671	***0.000***	26.52%

	Rain	-0.011	0.006	1.808	0.071	3.90%
	Sex	-0.212	0.124	1.689	0.091	2.19%
	Age class*Body mass	-0.007	0.004	1.621	0.104	9.27%
No. Vegetation Habitats (FR)	Intercept	-0.26	0.44	0.58	0.565	
	Body Mass	-0.65	0.19	3.29	***0.001***	45.79%
	Rain	-0.30	0.22	1.30	0.193	10.65%
	Number of GPS points	0.00	0.00	0.65	0.518	10.89%
	Body mass*rain	0.058	0.157	0.357	0.721	4.84%
No. Vegetation Habitats (ID)	Intercept	-0.48	0.51	0.90	0.366	
	Body Mass	-0.53	0.20	2.47	***0.014***	30.27%
	Number of GPS points	0.00	0.00	0.97	0.332	15.24%
Linear mixed effect models						
Nightly Dispersal	Intercept	0.10	0.14	0.72	0.473	
	Body mass	-0.33	0.13	2.57	***0.010***	43.44%
Distance: Roost to FA	Intercept	-0.01	0.11	0.09	0.931	
	Body mass	-0.30	0.10	2.95	***0.007***	33.40%
Distance: Between FA	Intercept	0.01	0.12	0.11	0.910	
	Body mass	-0.31	0.09	3.11	***0.005***	33.99%
Generalized linear mixed effect models						
No. Nightly FAs	Intercept	1.16	0.12	9.67	0.000	
	Body mass	-0.34	0.07	4.97	***0.000***	26.93%
	Number of GPS points	-0.02	0.01	0.48	0.222	0.12%
	Skeletal size	-0.04	0.02	0.36	0.330	0.02%

57

LITERATURE CITED

Andrews, C. W. 1900. A Monograph of Christmas Island (Indian Ocean). British Museum of Natural History.

Banack, S. A. 1998. Diet selection and resource use by flying foxes (Genus Pteropus). Ecology **79**:1949-1967.

Corbett, L., F. Crome, and G. Richards. 2003. Fauna survey of mine lease applications and National Park reference areas, Christmas Island. Christmas Island Phosphates, Perth.

James, D. J., G. J. Dale, K. Retallick, and K. Orchard. 2007. Christmas Island Flying-Fox *Pteropus natalis* Thomas 1887: An Assessment of Conservation Status and Threats. Page 55, Christmas Island National Park. Commonwealth of Australia.

Marshall, A. G. 1985. Old World phytophagous bats (Megachiroptera) and their food plants: A survey. Zoological Journal of Linnean Society **83**:169-351.

Nelson, S. L., M. A. Miller, E. J. Heske, and G. C. Fahey Jr. 2000. Nutritional quality of leaves and unripe fruit consumed as famine foods by the flying foxes of Samoa. Pacific Science **54**:301-311.

Orchard, K. 2006. Christmas Island flying-fox *Pteropus melanotus natalis*: Initial survey of population status and threat assessment. Unpub. rep. to Parks Australia North, Christmas Island.

Picot, M., R. Jenkins, O. Ramilijaona, P. A. Racey, and S. M. Carriere. 2007. The feeding ecology of *Eidolon dupreanum* (Pteropodidae) in eastern Madagascar. African Journal of Ecology **45**:645.

Tidemann, C. R. 1985. A study of the status, habitat requirements and management of the two species of bat on Christmas Island (Indian Ocean): Final report. Zoology Department, Australian National University, Canberra, Australia.

Wiles, G. J., and M. S. Fujita. 1992. Food plants and economic importance of flying foxes on Pacific islands. In Pacific Flying Foxes: Proceedings of an International Conference. U.S. Department of the Interior Fish and Wildlife Service Washington, DC 20240:24-35.
