# Supplementary material for: Body-size dependent foraging strategies in the Christmas Island flying-fox: implications for seed and pollen dispersal within a threatened island ecosystem
Source: Mov Ecol. 2022 Apr 11;10:19. doi: 10.1186/s40462-022-00315-8 (PMC8996557; doi:10.1186/s40462-022-00315-8)
Supplement: Supplementary file 2 — Additioanal file 2. Detailed results on observations of foraging resource defense by Christmas Island flying-foxes (Pteropus natalis). [file 40462_2022_315_MOESM2_ESM.pdf]

Additional File 2: Supplemental results

Foraging resource defense

Over the course of this study (August 2015 – November 2017) we conducted one to three-hour long surveys (*n* = 272), totaling 306 survey-hours, of flying-foxes at foraging sites. Out of the 272 surveys conducted, 105 surveys included an observation of *P. natalis* exhibiting behaviors consistent with resource defense. This included 47 observations in which a resident individual successfully defended a foraging resource against an intruder, and 58 observations in which an intruder aggressively displaced a resident.

The most frequent behavior observed was vocalization, which occurred in 71% (*n* = 74) of all interactions. Among observations in which a resident successfully defended a foraging resources from an intruder, vocalization alone occurred in 40% (n = 19) of confrontations. Wing spreading alone (*n* = 16), and vocalizations combined with wing spreading (*n* = 12), occurred in 34% and 25% of observed interactions.

Among observations in which an intruder successfully displaced a resident, fighting, which includes vocalizations occurred in 55% (*n* = 32) of confrontations and always resulted in the resident individual either being chased (28%; *n* = 16) or flying away (28%; *n* = 16). Vocalizations followed by a chase occurred in 21% (*n* = 12), and a chase with no vocalization occurred in 24% (*n* = 14) of confrontations.

Scent marking of branches, a behavior often observed among males defending a harem of females during the breeding season (C.M.T., pers., obs.), was observed once at a foraging site by an adult male *P. natalis*, during a capture session conducted at a feeding tree. During the early evening on May 5, 2017, five individuals were captured between 1540 – 1730 hr at a ripe *C. papaya* (supplementary material 2: Table S1). Each individual was captured shortly after landing

in the papaya tree with a landing net, leaving the ripe fruit to attract other individuals. Upon landing and prior to capture, the fifth individual, a large adult male with the highest body mass (478 g) of the five individuals caught, began to scent mark the branches of the tree. This individual was captured at 1730 hr, approximately 12 minutes before sunset, with ripe fruit still remaining in the tree. Over the course of the next hour (1730 – 1830 hr) four additional individuals entered the area and circled above the foraging tree several times without landing before leaving the area.

Table S2. Capture time, body mass and sex and age class for five Christmas island flying foxes (*Pteropus natalis*) captured at a fruiting papaya tree (*C. papaya*) on May 5, 2017.

Local Capture Time	Body Mass	Sex/Age class
15:40	409.00	♂ Sub-adult
16:33	398.00	♂ Adult
16:36	297.00	♂ Juvenile
17:06	325.00	♀ Sub-adult
17:30	478.00	♂ Adult
